# *Hdac3* deficiency limits periosteal reaction associated with Western diet feeding in female mice

**DOI:** 10.1111/jcmm.70081

**Published:** 2024-09-11

**Authors:** Elizabeth K. Vu, Ismael Y. Karkache, Anthony Pham, Jinsha Koroth, Elizabeth W. Bradley

**Affiliations:** ^1^ Department of Orthopedic Surgery Medical School, University of Minnesota Minneapolis MN USA; ^2^ Comparative Molecular Biosciences School of Veterinary Medicine St. Paul MN USA; ^3^ Stem Cell Institute, University of Minnesota Minneapolis MN USA

**Keywords:** Ccl2, high‐fat diet, macrophage, osteoblast, osteoclast

## Abstract

Diet‐induced obesity is associated with enhanced systemic inflammation that limits bone regeneration. HDAC inhibitors are currently being explored as anti‐inflammatory agents. Prior reports show that myeloid progenitor‐directed *Hdac3* ablation enhances intramembranous bone healing in female mice. In this study, we determined if *Hdac3* ablation increased intramembranous bone regeneration in mice fed a high‐fat/high‐sugar (HFD) diet. Micro‐CT analyses demonstrated that HFD‐feeding enhanced the formation of periosteal reaction tissue of control littermates, reflective of suboptimal bone healing. We confirmed enhanced bone volume within the defect of *Hdac3*‐ablated females and showed that *Hdac3* ablation reduced the amount of periosteal reaction tissue following HFD feeding. Osteoblasts cultured in a conditioned medium derived from *Hdac3*‐ablated cells exhibited a four‐fold increase in mineralization and enhanced osteogenic gene expression. We found that *Hdac3* ablation elevated the secretion of several chemokines, including CCL2. We then confirmed that *Hdac3* deficiency increased the expression of *Ccl2.* Lastly, we show that the proportion of CCL2‐positve cells within bone defects was significantly higher in *Hdac3‐*deficient mice and was further enhanced by HFD. Overall, our studies demonstrate that *Hdac3* deletion enhances intramembranous bone healing in a setting of diet‐induced obesity, possibly through increased production of CCL2 by macrophages within the defect.

## 1 INTRODUCTION

Intramembranous ossification, where bone forms directly from condensations of mesenchymal progenitor cells, helps to pattern many of the flat bones within the body. In addition to helping form the skeleton during development, recapitulation of this process aids during bone healing following injury as well as integration of dental and orthopaedic implants.^1^ This includes stress fracture healing and type I fracture healing achieved with rigid fixation.^2^ Moreover, intramembranous bone regeneration contributes in part to the healing of type II fractures that are also characterized by regions of cartilaginous callus formation.^2^ Several preclinical models of intramembranous bone regeneration simulate bone injuries that heal through intramembranous ossification, including the single cortex defect model.^3^ In this model, the recruitment of periosteal stem cells to the injury site generates bone‐forming osteoblasts within the defect.^4^ Although periosteal stem cells express chemokine receptors,^5^ the study of the mechanisms for recruitment and expansion of periosteal skeletal stem cells to the defect site during intramembranous bone regeneration and how this process is impacted by diet and/or inflammation is less characterized.

Macrophages aid in bone growth during development, promote bone formation^6^, ^7^, ^8^, ^9^, ^10^, ^11^, ^12^, ^13^, ^14^, ^15^, ^16^, ^17^, ^18^, ^19^ and facilitate bone healing during intramembranous bone regeneration.^6^, ^10^, ^20^, ^21^, ^22^ Broad depletion of macrophages, via genetic targeting or clodronate liposomes, attenuates intramembranous bone regeneration.^6^ Conversely, M‐CSF administration enhances the number of macrophages and intramembranous bone formation at the injury site.^6^ While optimal bone regeneration requires the action of macrophages, the molecular mechanisms by which macrophages promote intramembranous bone healing are less defined.

Osteoclasts likewise play a crucial role during bone healing. Detailed studies of endochondral fracture healing demonstrate that osteoclasts participate in both the early and late phases of fracture healing.^23^, ^24^ Recent evidence also documents that osteoclasts participating in endochondral fracture healing originate from circulating monocytes and/or erythro‐myeloid progenitors derived from the spleen.^25^, ^26^ Osteoclasts are likewise present throughout woven bone during intramembranous bone healing of cortical defects.^6^ In contrast to endochondral fracture healing, OPG‐mediated clearance of osteoclasts during intramembranous bone healing reduced osteoclast number and resorption but did not limit the formation of woven bone within the defect site.^6^

In the US and other developed countries, consumption of a western diet (e.g. high‐fat, high‐refined sugar) is linked to adverse health outcomes such as obesity, diabetes and cardiovascular events.^27^ The effects of a western diet on bone health are complex, but several pre‐clinical studies suggest that a high‐fat/high‐sugar diet limits bone regeneration and fracture healing.^28^, ^29^, ^30^ Moreover, the effects of a western diet on bone mass and fracture healing may be influenced by sex.^31^

Our prior work demonstrates that conditional deletion of *Hdac3* in myeloid progenitor cells enhances intramembranous bone healing in female mice fed a low‐fat/low‐refined sugar diet.^21^ Prior work also well establishes that genetic depletion of *Hdac3* in myeloid lineage cells limits inflammatory responses and limits osteoclastogenesis^21^, ^32^, ^33^, ^34^, ^35^, ^36^; however, these studies were also performed using mice fed a low‐fat/low‐sugar diet. In this study, we evaluated the effects of myeloid progenitor‐directed ablation of *Hdac3* during intramembranous bone regeneration in a model of western diet feeding.

## 2 MATERIALS AND METHODS

### 2.1 Generation of Hdac3 conditional knockout mice and diet feeding

*Hdac3* ^fl/fl^ mice on the C57Bl6/J background^37^ were crossed with mice expressing Cre‐recombinase under the control of the LysM promoter^38^ to delete *Hdac3* within Cre‐expressing cells. Mice were genotyped for Cre as previously described,^39^ or the *Hdac3* floxed allele using the following primers: A: 5′‐CCACTGGCTTCTCCTAAGTTC‐3′, B: 5′‐CCCAGGTTAGCTTTGAACTCT‐3′ and C: 5′‐TTTCCGTATTTGTGGAAGGA‐3′. *Hdac3*‐ablated animals arising from these crossings are referred to as *Hdac3* cKO_LysM_ mice. Littermate Cre^−^ animals from crossings were used as controls as appropriate. Animals were housed in an accredited facility under a 12‐h light/dark cycle. All animal research was conducted according to guidelines provided by the National Institute of Health and the Institute of Laboratory Animal Resources, National Research Council. The University of Minnesota Institutional Animal Care and Use Committee approved all animal studies.

Male and female mice were weaned at 4 weeks old onto either a standard diet or a high‐fat/high‐glucose diet (Research Diets D12492, 60% fat, 20% protein, 20% carbohydrate. New Brunswick, NJ). Numbers of males and females of each genotype within diet groups are indicated within figure legends.

### 2.2 Cortical bone defect generation

Single‐cortex, fully stabilized, mid‐diaphysis femoral defects were generated in the right femur as previously described.^3^, ^21^ Mice in all experimental groups were given Buprenorphine SR provided perioperatively at 0.5 mg/kg given 2–4 h before surgery via subcutaneous injection. Mice were then anaesthetised with isoflurane and prepared for aseptic surgery. A small incision was made in the skin overlying the right hamstring and the femoral bone shaft was exposed by blunt dissection of the underlying muscle. A 0.7 mm diameter steel burr drill bit (#19007‐07, Fine Science Tools, Foster City, CA) and an electric drill were used to induce a single‐cortex defect in the mid‐diaphysis of the femur. Defects were immediately irrigated with 1 mL sterile saline followed by incision closure. Mice were sacrificed by carbon dioxide inhalation on postoperative day 14.

### 2.3 Micro‐computed tomography of the bone defect site

Right femurs were collected 14 days following defect generation and fixed in 10% neutral buffered formalin for 24 h. Femurs were then stored in 70% ethanol prior to scanning using the XT H 225 micro‐computed tomography (micro‐CT) machine (Nikon Metrology Inc., Brighton, MI). Scans were performed at 120 kV, 61 μA and 720 projections at two frames per projection with an integration time of 708 ms as previously described.^40^ Scans were performed at an isometric voxel size of 7.11 μm with a 1‐mm aluminium filter, 17 minutes per scan. Each scan volume was reconstructed using CT Pro 3D (Nikon Metrology Inc.). Reconstructions were converted to bitmap data sets using VGStudio MAX 3.2 (Volume Graphics GmbH, Heidelberg, Germany). Scans were reoriented via DataViewer (SkyScan, Bruker micro‐CT, Kontich, Belgium) to create a new bitmap data set for consistent analysis. Morphometric analysis was performed using SkyScan CT Analyser (CTAn, Bruker micro‐CT, Belgium). Bruker's instructions and guidelines for analysis within the field were followed throughout the analysis.^40^ 3D analysis of the cortical bone at the defect site was performed in the femoral midshaft approximately 0.7 mm proximal to the growth plate and extending 1.5 mm proximally towards the bone diaphysis. The region of interest (ROI) was defined at the midpoint of the cortical defect, extending approximately 0.35 mm in both directions. The ROI was manually contoured around the cortical bone immediately surrounding the defect site, including the periosteum. Whole bone 3D analysis of this ROI was conducted following subsequent automated contouring. Binary selection of all samples resulted in a global threshold used to separate bone from surrounding tissue within the ROI. 3D models of the defect ROI were generated using 3D reconstruction software (CtVox, Bruker micro‐CT, Belgium) in a transverse orientation for qualitative analysis.

### 2.4 Bone histomorphometry

Femurs containing cortical bone defects were decalcified, paraffin‐embedded and sectioned to a thickness of 7 μm. Sections were TRAP stained using the ELF97 substrate (#387A‐1KT, Sigma‐Aldrich) and counterstained with DAPI. Serial sections were haematoxylin and eosin stained. The number of TRAP‐positive cells within the defect site and the number of osteoclasts per bone perimeter (No. Ocl/B.Pm.) within the defect and within periosteal reaction tissue were quantified as previously described. The percentage periosteal reaction tissue volume at the margins of the cortical defect per total tissue volume was evaluated using ImageJ.

### 2.5 Isolation and culture of bone marrow macrophages

Bone marrow macrophages (BMMs) were collected from 6‐ to 8‐week‐old *Hdac3* cKO_LysM_ females or their sex‐matched littermates and previously described.^21^, ^39^ Macrophages were cultured in the presence of 50 ng/mL M‐CSF (R and D Systems, 416‐ML‐050) and treated as per experimental design.

### 2.6 Osteoblast cell mineralization assays

Calvarial osteoblasts were collected from P7 male or female C57Bl6/J mice as previously described.^39^, ^41^ Osteoblasts were cultured (0.65 × 10^6^ cells/cm^2^) in a conditioned medium derived from macrophages isolated from *Hdac3* cKO_LysM_ or littermate control female mice that was diluted 1:1 in fresh α‐MEM and supplemented with 10% FBS, 50 μg/mL of ascorbate, 10 mm β‐glycerolphosphate and 1 × 10^7^ m dexamethasone. Cultures were fed every 3–4 days with a respective diluted conditioned medium plus osteogenic supplements. Cells were fixed and stained with Alizarin Red on day 10. For blocking experiments, the CCL2 neutralizing antibody (AF‐479‐NA, R&D Systems) was used at a concentration of 1 μg/mL.

### 2.7 Wound healing assays

ST2 osteoprogenitor cells were cultured at a density of 5 × 10^5^ cells/cm^2^ and incubated overnight. A scratch in each cell monolayer (*n* = 2/group) was made with a 10 μL pipette tip. Cells were then washed twice with PBS and placed in a control or *Hdac3* cKO‐conditioned medium for 12 h and fixed with neutral buffered formalin. Images were digitally collected using phase contrast microscopy and the area of each wound was assessed using ImageJ software.

### 2.8 RNA extraction and semi‐quantitative PCR

Total RNA was extracted from primary BMMs or calvarial osteoblasts using TRIzol (Invitrogen) and chloroform, and 1 μg was reverse transcribed using the SuperScript III first‐strand synthesis system (Invitrogen). The resulting cDNAs were used to assay gene expression via real‐time PCR using the gene‐specific primers listed in Table 1. Fold changes in gene expression for each sample were calculated using the 2^−ΔΔCt^ method relative to control after normalization of gene‐specific C_t_ values to Ywhaz C_t_ values.^21^, ^39^ Shown are data from three replicates per group of each genotype with three independent experiments.

**TABLE 1:** qPCR primers.

Gene	Forward primer	Reverse primer
*Alpl*	5′‐CAGGCCGCCTTCATAAGCA‐3′	5′‐CAGGCCGCCTTCATAAGCA‐3′
*Bgn*	5′‐GAGACCCTGAATGAACTCCACC‐3′	5′‐CTCCCGTTCTCGATCATCCTG‐3′
*Col1a1*	5′‐GCTTCACCTACAGCACCCTTGT‐3′	5′‐TGACTGTCTTGCCCCAAGTTC‐3′
*Ccl2*	5′‐GATGCAGTTAACGCCCCACT‐3′	5′‐GACCTTGGTGACAAAAACTACAGC‐3′
*Dlx5*	5′‐AGCTACGCTAGCTCCCTACCACC‐3′	5′‐AGCTACGCTAGCTCCCTACCACC‐3′
*Hdac3*	5′‐CCCGCATCGAGAATCAGAAC‐3′	5′‐TCAAAGATTGTCTGGCGGATCT‐3′
*Ibsp*	5′‐GAATGGCCTGTGCTTTCTCG‐3′	5′‐CCGGTACTTAAAGACCCCGTT‐3′
*Sp7*	5′‐GGAGGTTTCACTCCATTCCA‐3′	5′‐TAGAAGGAGCAGGGGACAGA‐3′
*Ywhaz*	5′‐GCCCTAAATGGTCTGTCACC‐3′	5′‐GCTTTGGGTGTGACTTAGCC‐3′

### 2.9 Cytokine arrays

Bone marrow macrophages were collected from 6‐ to 8‐week‐old *Hdac3* cKO_LysM_ females or their sex‐matched littermates. Macrophages were cultured in the presence of 50 ng/mL M‐CSF (R and D Systems, 416‐ML‐050, Minneapolis, MN) for 3 days and a conditioned medium was collected. Cytokine arrays using the Proteome Profiler Mouse XL Cytokine Array (R and D Systems, ARY028) were then performed in duplicate according to the manufacturer's specifications using the conditioned medium. Spot intensities were determined using ImageJ software and the average intensity was determined. Heatmaps of differentially secreted cytokines were generated using Morpheus analysis software (Broad Institute).

### 2.10 Immunohistochemistry

For immunohistochemical staining, we utilized the Mouse‐ and Rabbit‐specific HRP/DAB (ABC) IHC Detection (Abcam, #ab236466) according to the manufacturer's specifications. Briefly, sections were deparaffinized in xylenes and a series of graded ethanol and rehydrated in water. Sections were blocked and a primary antibody directed towards CCL2 (ThermoFisher Scientific, #MA5‐17040) or an irrelevant control was applied and incubated overnight at 4°C. Streptavidin polyvalent secondary antibodies and biotin‐linked horseradish peroxidase (HRP) were then applied and chromogens were developed with 3,3′‐diaminobenzidine. Sections were then briefly counterstained with Fast Green, dehydrated and coverslips were applied with a resinous medium. Digital images of the defect site were collected and the number of positively staining cells per tissue area was determined using ImageJ software.

### 2.11 Statistics

Statistics were performed in GraphPad Prism (Version 9) using Student's *t* test or one‐way ANOVA as appropriate and post‐hoc tests for multiple comparisons when necessary. Specific *p* values under 0.1 are shown within the figures. Data are shown as box plots from the 25th to 75th percentiles, with whiskers extending to the minimum and maximum value and means shown by horizontal lines or means ± standard deviation.

## 3 RESULTS

### 3.1 *Hdac3* ablation limits periosteal reaction in females following western diet feeding

**FIGURE 1:**
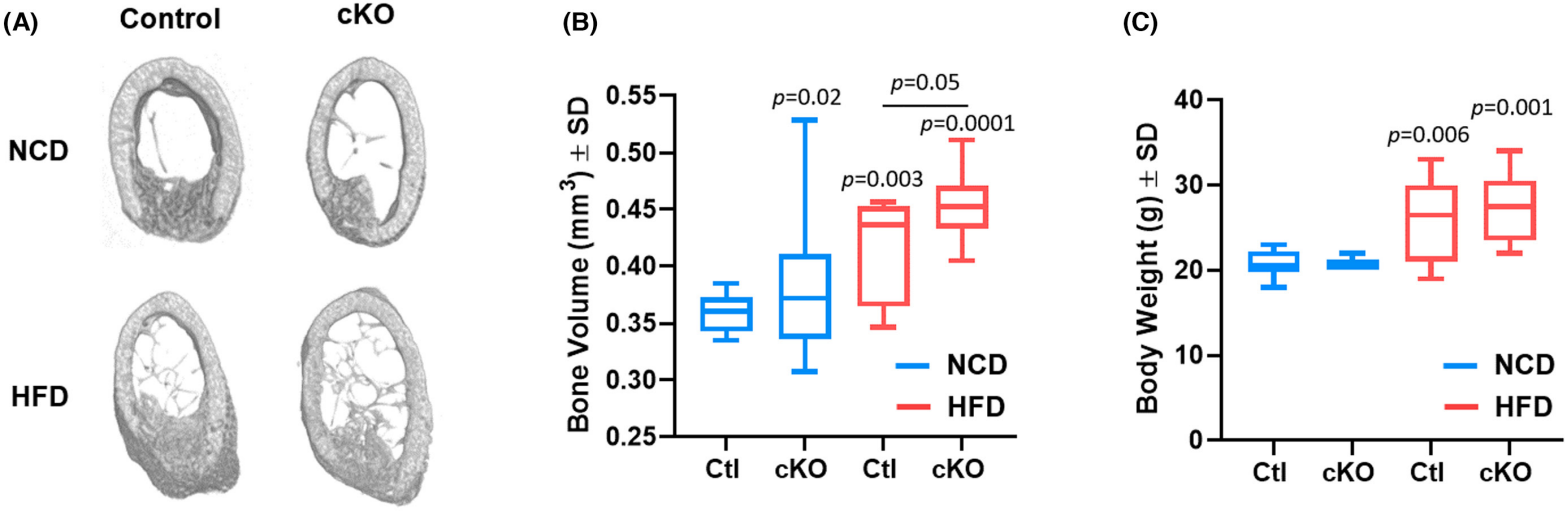
*Hdac3* ablation enhances intramembranous bone healing of female mice fed a western diet. *Hdac3* cKO_LysM_ females and their sex‐matched littermate controls were fed a high‐fat/high‐sugar diet (HFD, Control: *n* = 10, *Hdac3* cKO: *n* = 9) or normal chow (NCD, Control: *n* = 10, *Hdac3* cKO: *n* = 9) following weaning. Mice were aged to 12 weeks old and single cortex defects were generated in the femoral mid‐shaft. Two weeks following defect generation, micro‐CT analyses were performed. Shown in (A) are reconstructions for femoral cortical defects. (B) Bone volume within the defect, *p* values as shown. (C) Body weights, *p* values as shown.

*Hdac3*‐ablated male and female mice and their control littermates were randomly assigned to either a normal chow diet (NCD) or a high‐fat/high‐sugar diet (HFD) after weaning. We then generated single cortex, fully stabilized bone defects within the right femoral mid‐diaphysis of 12‐week‐old mice. Micro‐CT analyses of the bone defect of female mice 14 days after defect generation revealed that *Hdac3* ablation enhanced bone volume at the defect site when comparing cKO (conditional knock out) and littermate control NCD‐fed females (Figure 1A,B), confirming our prior published observations.^21^ Likewise, HFD‐fed controls also demonstrated increased bone volume within the defect compared to NCD‐fed control mice (Figure 1A,B). *Hdac3* ablation enhanced this effect (Figure 1A,B). We confirmed that a high‐fat/high‐sugar diet feeding increased the body mass of both control and *Hdac3*‐ablated females (Figure 1C). No differences in body weight were observed between control and *Hdac3*‐ablated female animals fed a high‐fat/high‐sugar diet (Figure 1C).

**FIGURE 2:**
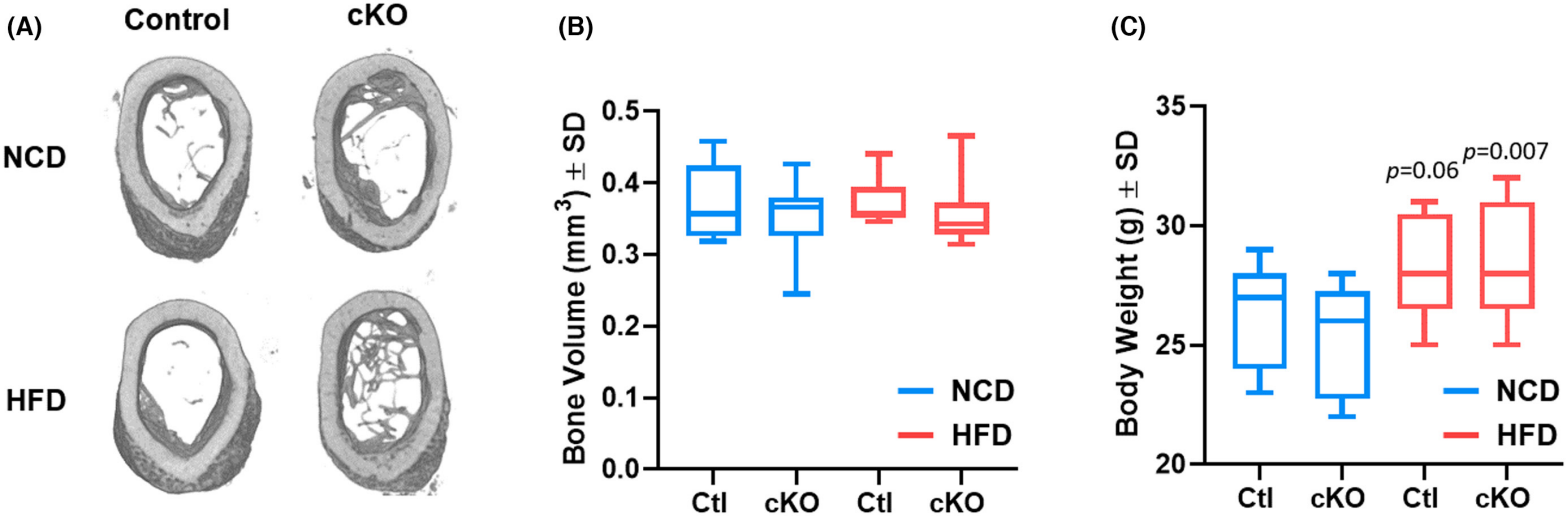
Male mice did not exhibit altered intramembranous bone healing. *Hdac3* cKO_LysM_ males and their sex‐matched littermate controls were fed a high‐fat/high‐sugar diet (HFD, Control: *n* = 9, *Hdac3* cKO: *n* = 10) or normal chow (NCD, Control: *n* = 9, *Hdac3* cKO: *n* = 10) following weaning. Mice were aged to 12 weeks old and single cortex defects were generated in the femoral mid‐shaft. Two weeks following defect generation, micro‐CT analyses were performed. Shown in (A) are reconstructions for femoral cortical defects. (B) Bone volume within the defect. (C) Body weights, *p* values as shown.

Our prior report documents that enhanced bone healing of *Hdac3*‐ablated mice was observed in females, but not males. We confirmed that observation of male mice fed either normal chow or a high‐fat/high‐sugar diet (Figure 2A,B). We verified that high‐fat/high‐sugar diet feeding increased body mass of both control and *Hdac3*‐ablated males (Figure 2C). No differences were observed between control and *Hdac3*‐ablated male animals fed a high‐fat/high‐sugar diet.

**FIGURE 3:**
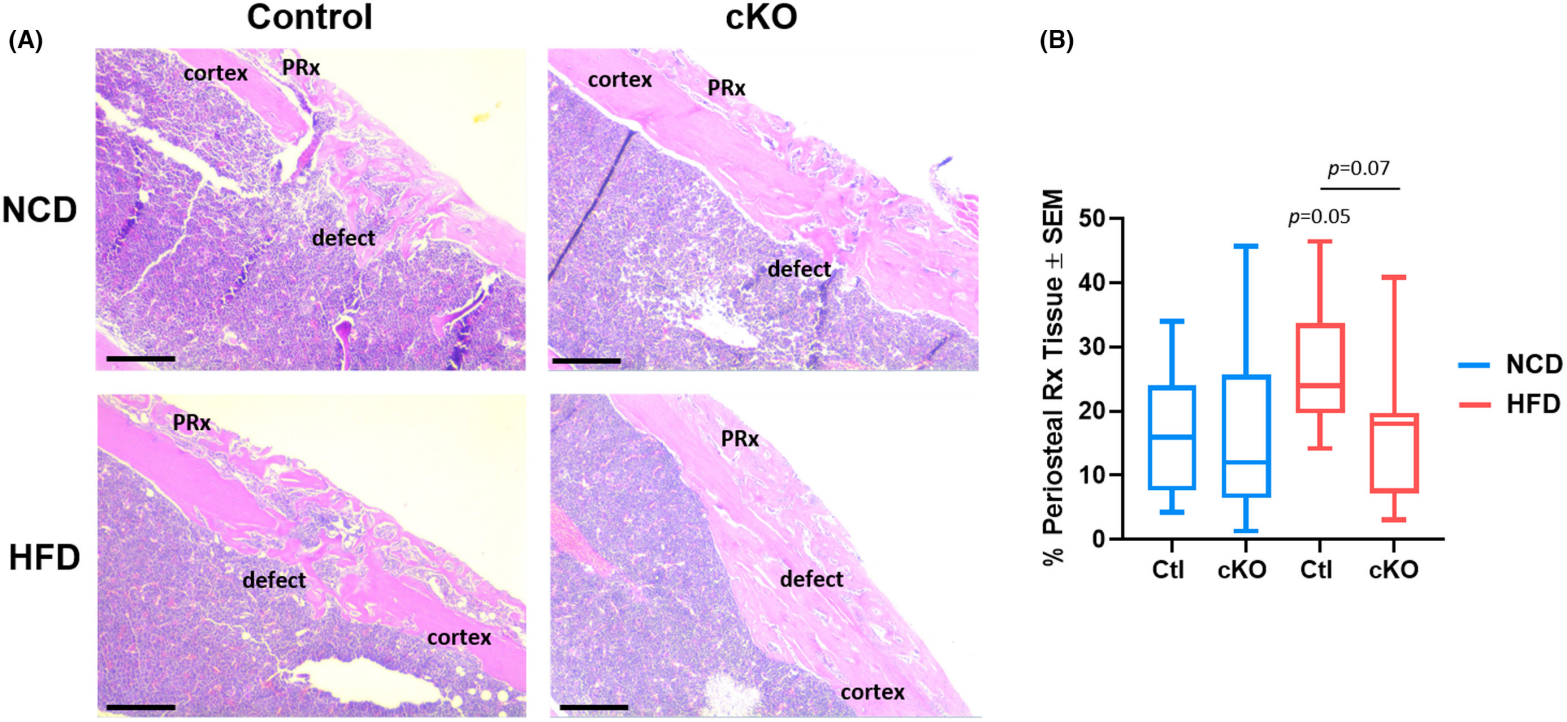
*Hdac3* ablation limits periosteal reaction in female mice fed a western diet. *Hdac3* cKO_LysM_ females and their sex‐matched littermate controls were fed a high‐fat/high‐sugar diet (HFD, Control: *n* = 5, *Hdac3* cKO: *n* = 5) or normal chow (NCD, Control: *n* = 5, *Hdac3* cKO: *n* = 5)) following weaning. Mice were aged to 12 weeks old and single cortex defects were generated in the femoral mid‐shaft. Two weeks following defect generation, paraffin sections of the femoral defect were collected. (A) Hematoxylin and eosin staining of femoral defects was performed and (B) the percentage of periosteal tissue per defect tissue area was determined. Specific *p* values are as shown. Scale bar is 100 μm.

**FIGURE 4:**
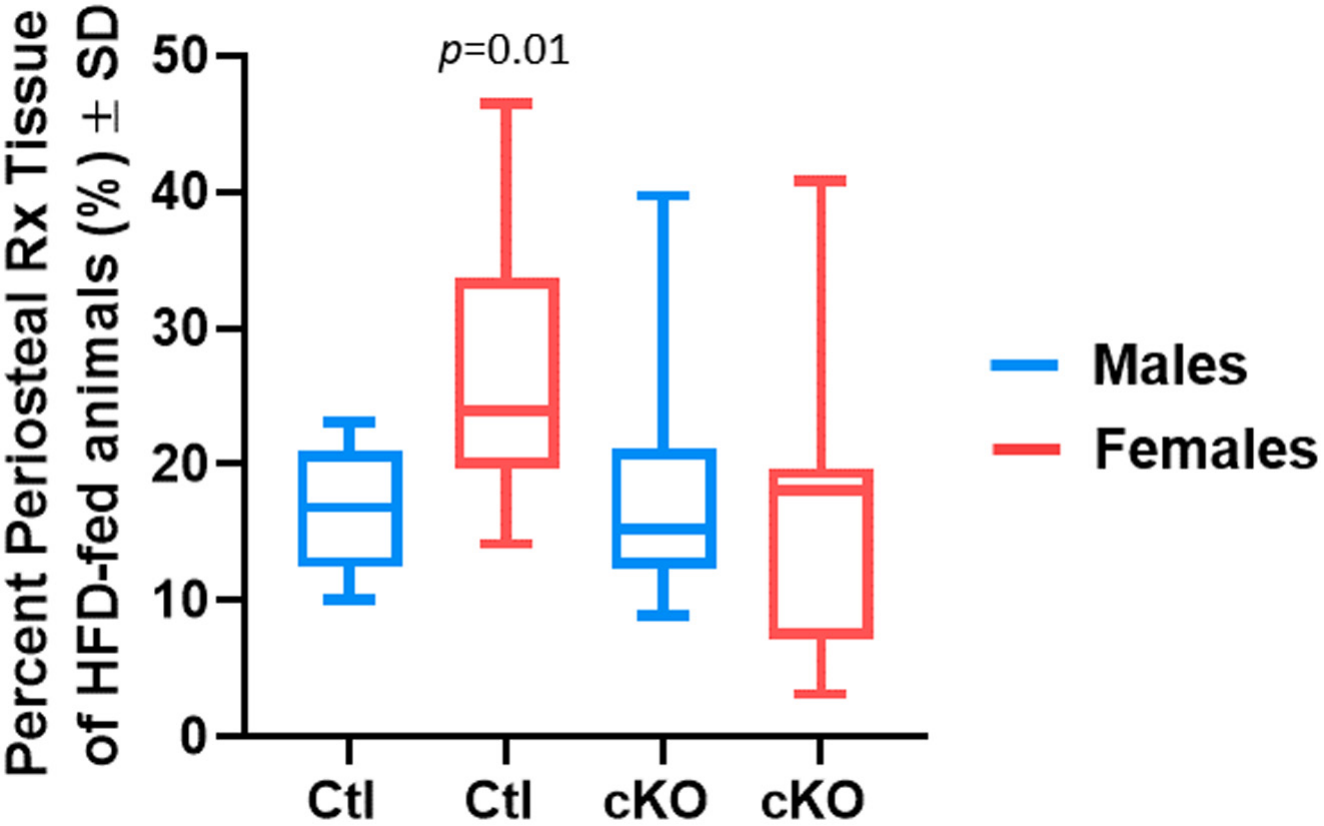
HFD‐fed females form significant periosteal reaction tissues following injury, but males do not. *Hdac3* cKO_LysM_ males and females and their sex‐matched littermate controls were fed a high‐fat/high‐sugar diet (HFD, Control: *n* = 10, *Hdac3* cKO: *n* = 9–10) following weaning. Mice were aged to 12 weeks old and single cortex defects were generated in the femoral mid‐shaft. Two weeks following defect generation, micro‐CT analyses were performed and the percentage of periosteal bone volume was determined, *p* values as shown.

We noted enhanced bone formation along the periosteal surface of high‐fat/high‐sugar diet‐fed female mice in micro‐CT reconstructions. Therefore, we first quantified the amount bone formed along the periosteal surface. Femurs containing cortical bone defects were decalcified, paraffin‐embedded and sectioned to a thickness of 7 μm and haematoxylin/eosin stained. We observed enhanced bone formation along the periosteal surface (e.g. periosteal reaction) of control high‐fat/high‐sugar‐fed mice compared to littermates fed a normal chow diet (Figure 3A,B). Ablation of *Hdac3* did not affect the amount of periosteal reaction in normal chow‐fed mice but did limit the amount of bone formed along the periosteal surface of high‐fat/high sugar‐fed animals (Figure 3A,B). We also assessed the amount of periosteal reaction tissue in males and females fed an HFD by removing the amount of bone volume within the defect from the perioseal reaction tissue in our micro‐CT analyses (Figure 4). No difference in the percentage of periosteal reaction tissue was noted between control and *Hdac3* cKO males (Figure 4). In contrast, control females demonstrated increased periosteal reaction tissue compared to control males (Figure 4). This was mitigated by *Hdac3* ablation in females. These data suggest that myeloid‐progenitor‐directed deletion of *Hdac3* limits unfavourable bone formation in female mice fed a western diet.

**FIGURE 5:**
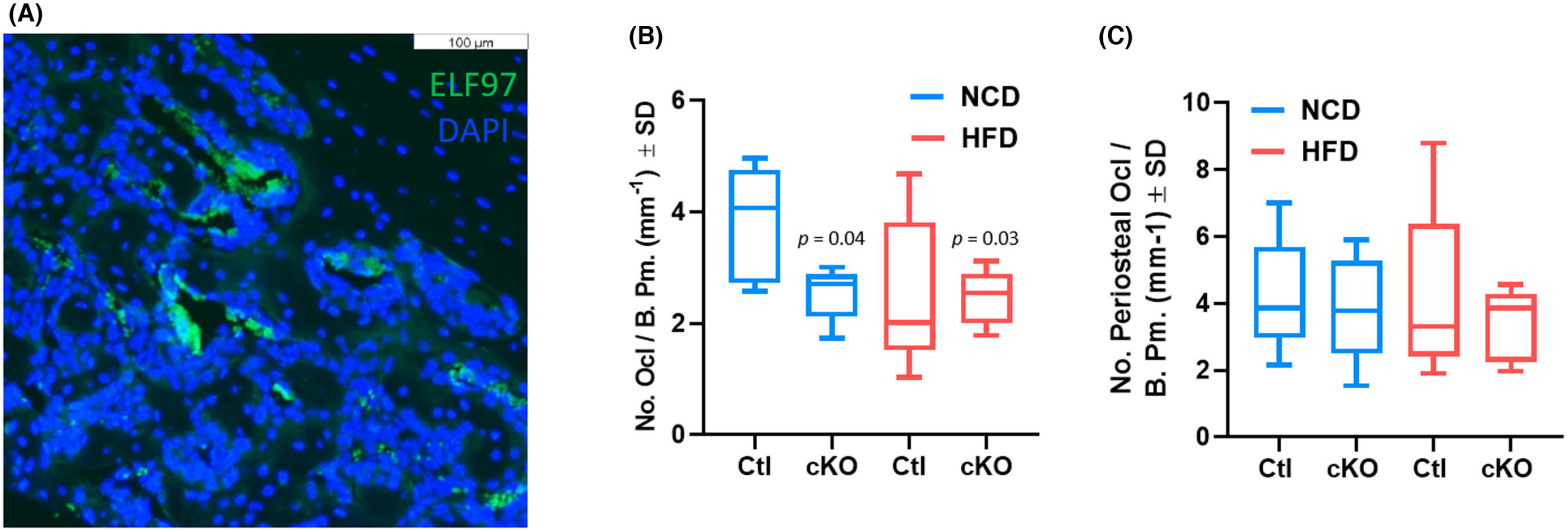
Western diet feeding does not alter osteoclast number with intramembranous bone defects. *Hdac3* cKO_LysM_ females and their sex‐matched littermate controls were fed a high‐fat/high‐sugar diet (HFD, Control: *n* = 5, *Hdac3* cKO: *n* = 5) or normal chow (NCD, Control: *n* = 5, *Hdac3* cKO: *n* = 5)) following weaning. Mice were aged to 12 weeks old and single cortex defects were generated in the femoral mid‐shaft. Two weeks following defect generation, paraffin sections of the femoral defect were collected and ELF97/DAPI staining was performed. (A) Representative ELF97 staining within the femoral defect, *p* values are as shown. (B) Quantification of ELF97‐postitive osteoclasts per bone perimeter (No. Ocl/B.Pm.) within the femoral defect. (C) Quantification of ELF97‐postitive osteoclasts per bone perimeter (No. Ocl/B.Pm.) within periosteal reaction tissue.

We next quantified the number of multinucleated ELF97‐positive cells (e.g. osteoclasts) within the defect site and within periosteal reaction tissue. Paraffin sections were TRAP stained using the ELF97 substrate and counterstained with DAPI to visualize multinucleated osteoclasts. We confirmed that *Hdac3* ablation limits osteoclast number within the defect site when mice are fed either a normal or a high‐fat/high‐sugar diet (Figure 5A,B). High‐fat/high‐sugar diet did not change the number of osteoclasts within the defect of littermate control mice (Figure 5B). Likewise, no change in the number of osteoclasts within periosteal reaction tissue was noted within any group (Figure 5C).

### 3.2 Macrophages from *Hdac3*‐ablated females produce secreted factors that enhance osteoblast mineralization

**FIGURE 6:**
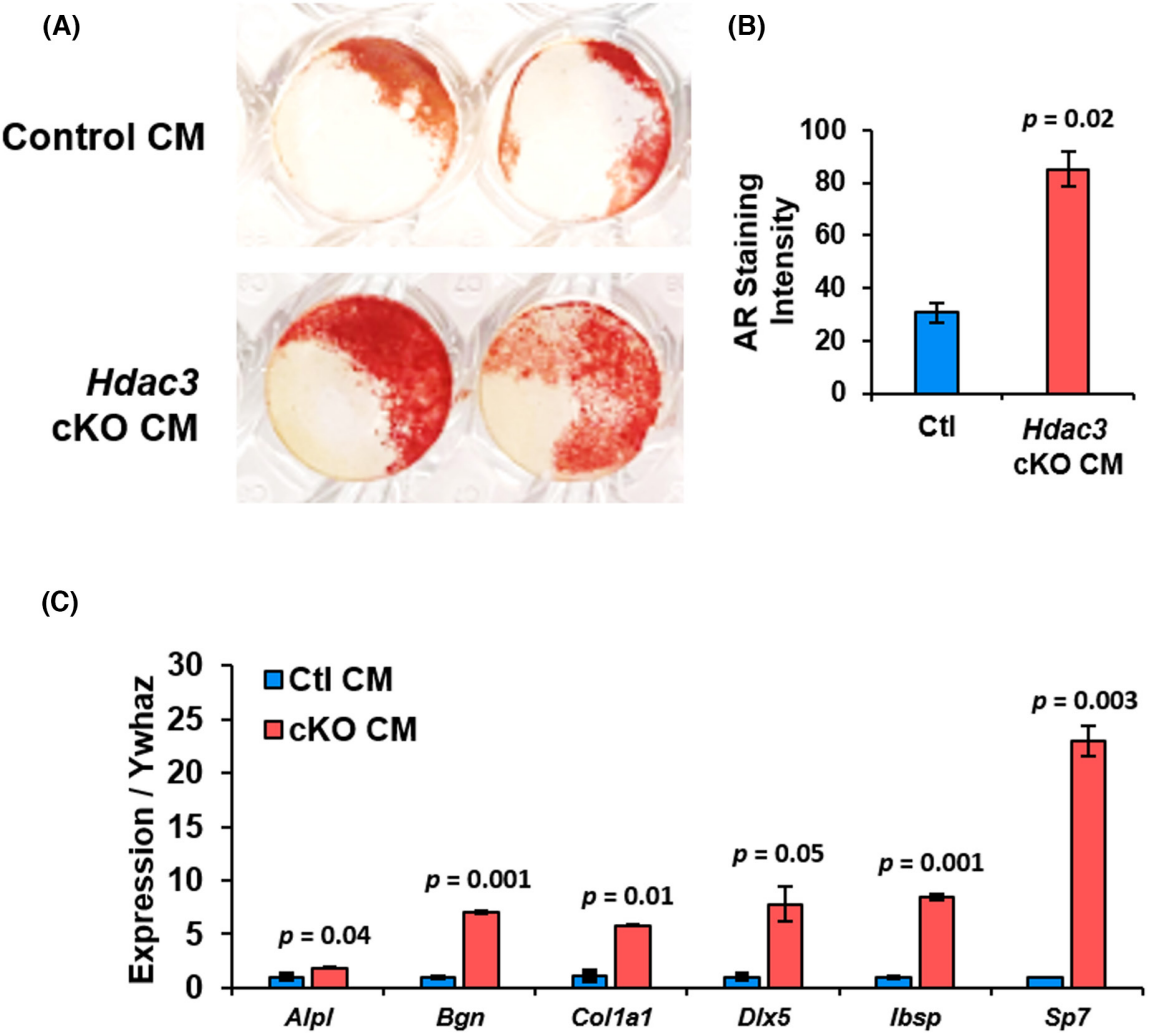
Macrophages isolated from *Hdac3*‐ablated females produce soluble factors that enhance osteoblast mineralization. Bone marrow macrophages (BMMs) were collected from 12‐week‐old female *Hdac3* cKO_LysM_ and control littermates. Cells were cultured in the presence of 50 ng/mL of M‐CSF for 4 days and conditioned medium (CM) was collected. WT calvarial osteoblasts were cultured in CM derived from female *Hdac3* cKO_LysM_ or control littermate BMMs. (A) Alizarin Red staining was performed and (B) Quantified, *p* value as shown, *n* = 3. (C) Expression of *Alpl*, *Bgn*, *Col1a1*, *Ibsp*, and *Sp7* were evaluated qPCR, *p* values as shown, *n* = 3.

Next, we explored mechanisms to explain how myeloid‐progenitor‐directed *Hdac3* ablation acts to enhance bone formation. We collected BMMs from 6‐ to 8‐week‐old *Hdac3*‐ablated mice and their control littermates. Bone marrow macrophages were cultured in the presence of macrophage colony‐stimulating factor (M‐CSF) for 3 days to induce macrophage differentiation. Cells were refed with medium containing M‐CSF and the conditioned medium was collected after 3 days. Calvarial osteoblasts from C57Bl/6 mice were then collected and cultured in macrophage‐conditioned medium for 7 days. Alizarin red staining of calvarial osteoblast cultures demonstrated that soluble factors produced by *Hdac3*‐ablated macrophages enhanced mineralization (+60%, Figure 6A,B). Moreover, a conditioned medium from *Hdac3*‐deficient macrophages enhanced expression of osteoblast phenotypic genes [e.g. alkaline phosphatase (*Alpl*), biglycan (*Bgn*), Type I Collagen (*Col1a1*), Distal‐less homeobox 5 (*Dlx5*), bone sialoprotein 2 (*Ibsp*) and Osterix (*Sp7*), Figure 6C].

### 3.3 *Hdac3* deficiency enhances chemokine secretion

**FIGURE 7:**
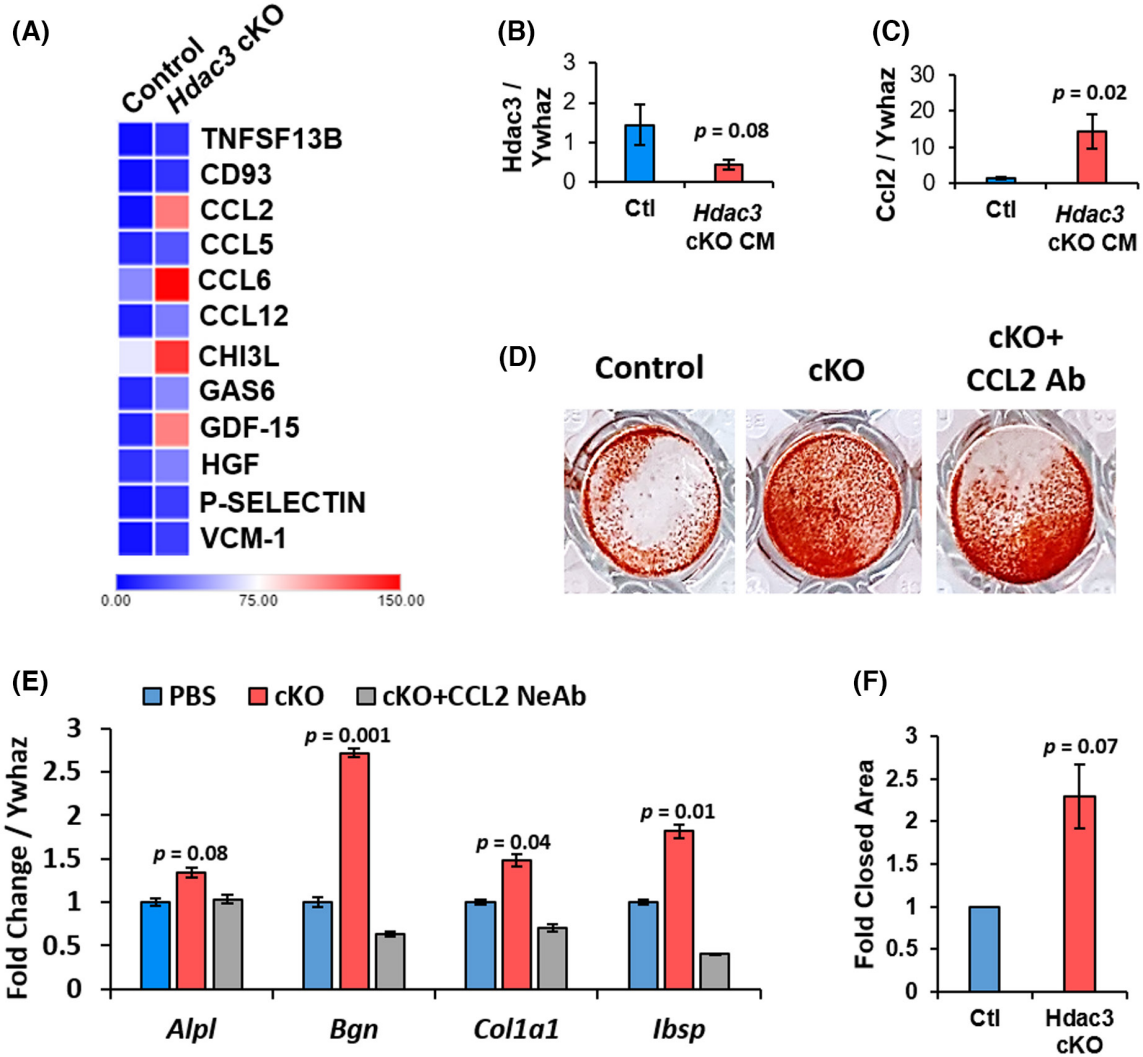
*Hdac3* deficiency increases the secretion of CCL2 by macrophages to enhance bone formation. Bone marrow macrophages (BMMs) were collected from 12‐week‐old female *Hdac3* cKO_LysM_ and control littermates. Cells were cultured in the presence of 50 ng/mL of M‐CSF for 4 days, and a conditioned medium (CM) was collected. (A) Heatmap of secreted cytokines from *Hdac3* cKO_LysM_ and control BMMs. Expression of (B) *Hdac3* and (C) *Ccl2* by BMMs was evaluated by qPCR, *p* values as shown, *n* = 3. (D) WT calvarial osteoblasts were cultured in CM derived from female *Hdac3* cKO_LysM_ or littermate control BMMs in the presence and absence of a CCL2 neutralizing antibody, and (E) gene expression of osteoblast markers was evaluated by qPCR, *p* values as shown, *n* = 3. (F) ST2 osteoprogenitor cells were cultured in CM derived from female *Hdac3* cKO_LysM_ or littermate control BMMs and wound healing assays were performed, *p* values as shown.

To identify potential soluble factors mediating these effects, we performed a cytokine array with a conditioned medium generated from *Hdac3* deleted macrophages and controls. As expected from prior reports, we confirmed that *Hdac3* enhances the secretion of CHI3L (Chitinase 3 like 1) by macrophages (Figure 7A). In addition, we demonstrate that the chemokines CCL2 (C‐C chemokine ligand) and CCL6, as well as GDF‐15 (growth and differentiation factor‐15) are enriched in the conditioned medium from *Hdac3*‐ablated macrophages (Figure 7A). We confirmed by qPCR that *Ccl2* expression is elevated by *Hdac3*‐deficient macrophages (Figure 7B,C).

### 3.4 CCL2 neutralization limits the effects of *Hdac3* deletion

We then examined the requirement of secreted CCL2 to mediate enhanced bone formation induced by a conditioned medium derived from *Hdac3*‐ablated macrophages. Although a conditioned medium from *Hdac3*‐ablated macrophages enhanced Alizarin Red staining of calvarial osteoblasts, this was attenuated through co‐treatment with a CCL2 neutralizing antibody (Figure 7D). Likewise, increased expression of *Alpl*, *Bgn*, *Col1a1* and *Ibsp* was blocked by neutralization of CCL2 within the conditioned medium of *Hdac3*‐ablated macrophages (Figure 7E). Lastly, we assayed migration of the osteoprogenitor ST2 cell line and found that a conditioned medium derived from *Hdac3*‐ablated macrophages increased wound healing compared to control a macrophage‐conditioned medium (Figure 7F).

### 3.5 Deletion of *Hdac3* elevates Ccl2 expression within the defect

**FIGURE 8:**
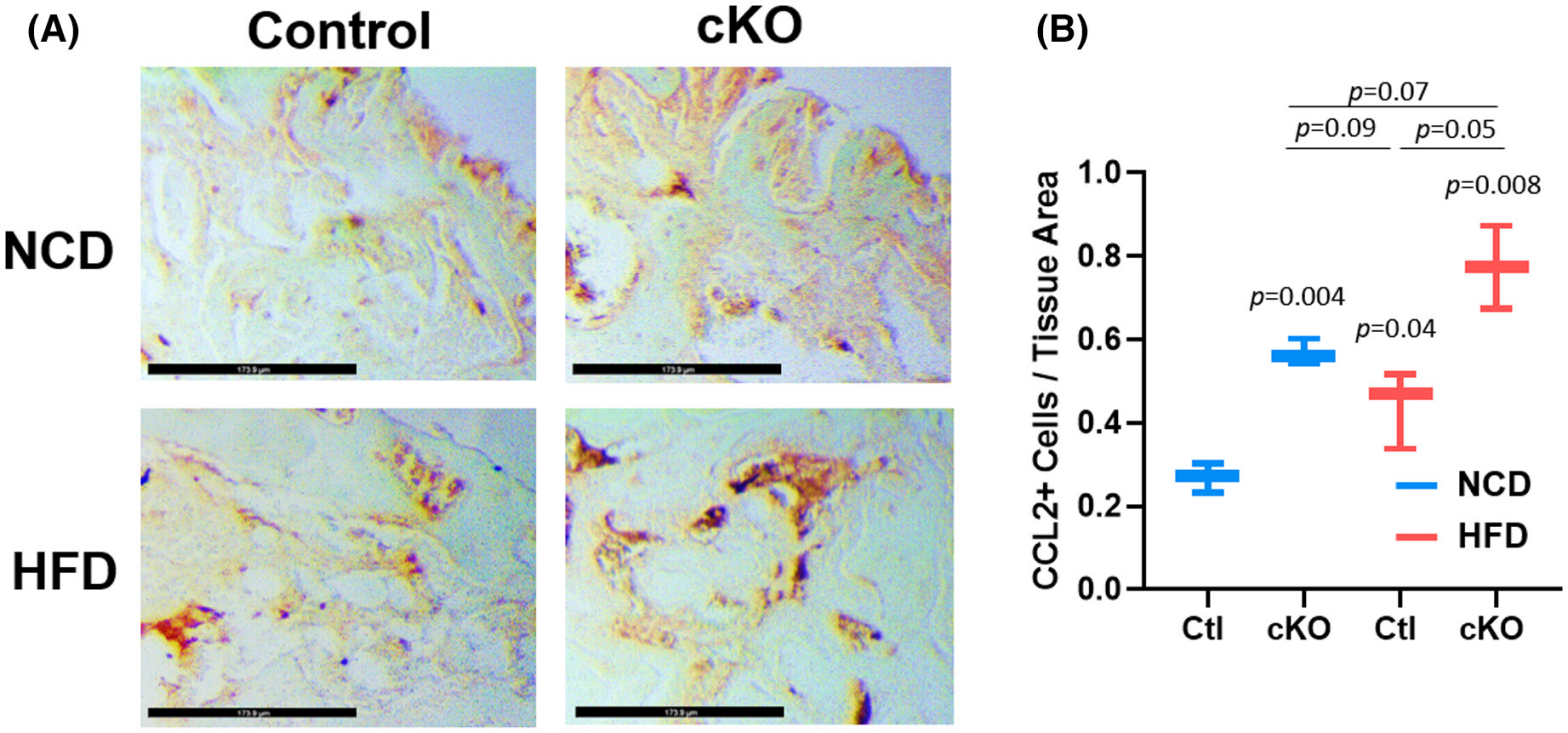
*Hdac3* deficiency increases CCL2 levels within intramembranous bone defects. *Hdac3* cKO_LysM_ females and their sex‐matched littermate controls were fed a high‐fat/high‐sugar diet (HFD) or normal chow (NCD) following weaning. Mice were aged to 12‐week‐old and single cortex defects were generated in the femoral mid‐shaft. Two weeks following defect generation, paraffin sections of the femoral defect were collected and (A) immunohistochemistry using and an anti‐CCL2 antibody was performed. The scale bar is 174 μm. (B) The number of CCL2‐expressing cells per tissue area was determined, *p* values as shown, *n* = 3/group.

We observed that *Hdac3*‐ablated macrophages produced higher levels of CCL2 and that CCL2 was required for enhanced mineralization of osteoblasts cultured in conditioned medium derived from *Hdac3*‐ablated cells. To determine if this may be true in vivo, we performed immunohistochemistry for CCL2 within the defect site of *Hdac3*‐ablated females and their control littermates. In animals fed a normal chow or high‐fat/high‐sugar diet, *Hdac3* ablation enhanced the number of CCL2‐positive cells per tissue area (Figure 8A,B). High‐fat/high‐sugar diet also increased numbers of CCL2‐expressing cells within the defect site, which was further enhanced by *Hdac3* ablation (Figure 8A,B). These data support that myeloid progenitor directed deletion of *Hdac3* enhances CCL2 expression in vivo.

## 4 DISCUSSION

In the work described here, we evaluated the effects of *Hdac3* conditional deletion within myeloid progenitors during intramembranous bone healing in the setting of western diet feeding. We found that high‐fat/high‐sugar diet feeding enhanced unfavourable bone formation (e.g. periosteal reaction) after injury; thus, *Hdac3* ablation limited the amount of periosteal reaction tissue in female mice, but males did not show any enhanced bone regeneration. This was accompanied by decreased numbers of osteoclasts within the defect site of *Hdac3*‐ablated females, but not within the periosteal tissue. Mechanistically, we show that *Hdac3*‐ablated macrophages produced CCL2 that is required to promote mineralization.

Chemokines are a large family of small, secreted proteins best known to promote chemotaxis and leukocyte infiltration; thus, they play an important role in morphogenesis and wound healing. CCL2 is a member of the CC‐type chemokines. Many cell types, including osteoblasts and various tissue‐resident macrophages, express CCL2 to induce monocyte chemotaxis. Genetic depletion of *Ccl2* in mice disrupts physiological bone remodelling, resulting in elevated bone mass and diminished osteoclast number.^42^, ^43^ In contrast, CCL2 facilitates the anabolic actions of intermittent PTH via increasing osteoblast number/activity and the catabolic actions of continuous PTH by enhancing bone resorption.^44^ CCL2 mediates signalling through the G protein‐coupled receptor CCR2. Prior studies have likewise documented that genetic depletion of *Ccr2* (e.g. *Ccr2* null mice) reduces the infiltration of macrophages and osteoclasts to fracture callus to limit endochondral fracture healing; furthermore, *Ccr2* ablation controls osteoclast activity in an estradiol (E2)‐dependent fashion.^45^ Although prior work describes the actions of *Ccl2* in physiological bone remodelling and PTH‐induced bone formation, the role of CCL2 during intramembranous bone healing is undefined. Periosteal stem cells express Ccr2,^46^ so the enhanced expression of Ccl2 by *Hdac3* ablated cells within the defect may promote the recruitment of stem cells to the defect site.

We observed increased bone volume within the defect when control mice were fed HFD chow. At first glance, this seemed contrary to published studies suggesting that HFD‐feeding attenuates bone regeneration.^28^, ^29^, ^30^ A closer examination of our data revealed that consumption of a high‐fat/high‐sugar diet increased the percentage of periosteal reaction within control female HFD‐fed mice. These data likewise support that HFD feeding diminishes bone regeneration via suboptimal bone healing. *Hdac3* ablation limited this response to high‐fat/high‐sugar feeding. These effects were limited to female mice.

Oestrogen receptors (ER) α‐ and β‐mediate genomic responses to estrogens. HDAC3 regulates responses to estrogens in a cell‐type‐specific manner. For instance, while one report demonstrated that *Hdac3* ablation promoted degradation of *Esr1* mRNA (i.e. the gene encoding ERα) in breast cancer cells,^47^ others have shown that HDAC3 inhibition promotes *Esr1* expression.^48^ Moreover, HDAC inhibitors and *Hdac3* ablation regulate the transcriptional activity of ERα and ERβ in a cell‐specific fashion.^49^, ^50^, ^51^, ^52^, ^53^ Myeloid cells express both ER isoforms, and the role of each respective receptor in controlling macrophage activities is not well established.^54^, ^55^, ^56^

*Hdac3* ablation in males does not alter bone healing in our prior published studies,^21^ and high‐fat/high‐sugar feeding did not alter this observation. The effects of *Hdac3* ablation could be driven by oestrogen‐dependent mechanisms, limiting the effects within males. Alternatively, other isoforms could be functionally compensating for *Hdac3* deficiency in males as has been observed in other organ systems.^57^, ^58^

Our study has several important limitations. We chose the LysM‐Cre driver to target myeloid progenitor cells due to the broad coverage of cell types involved in bone regeneration, including osteoclasts, macrophages and neutrophils. Prior studies likewise demonstrated that LysM‐Cre‐mediated deletion of *Hdac3* suppressed inflammatory responses and increased regeneration of other tissue types.^32^, ^33^, ^59^ In contrast, using this approach does not allow us to define the specific cells mediating the enhanced bone healing observed with targeted deletion of *Hdac3*. We utilized BMMs as our cell source, but isolation of specific macrophage subsets such as osteomacs may refine our understanding of how *Hdac3* limits bone healing within females. While mice within our study exhibited significant gains in weight and demonstrated corresponding decreases in weight adjusted cortical bone, we did not observe changes to glucose and insulin responsiveness in tolerance testing. This is in line with prior reports documenting that mice are resistant to the development of diabetes when placed on high‐fat/high‐sugar diet. This limitation limits our ability to accurately reflect the full spectrum of changes associated with consumption of a western diet in humans.

In this study, we confirmed that myeloid progenitor‐directed ablation of *Hdac3* enhances intramembranous bone regeneration in females, but not males. Moreover, we found that *Hdac3* deletion protected against unfavourable bone healing associated with consumption of a high‐fat/high‐sugar diet. We also found that *Hdac3*‐deleted macrophages secreted factors that enhanced mineralization, including CCL2; thus, *Hdac3* deficiency may limit periosteal reaction following injury due to increased CCL2‐mediated recruitment of periosteal stem cells to the defect site. Periosteal reaction tissue is often disorganized and of lower quality,^30^, ^60^ so strategies to limit this type of unfavourable bone formation are significant. Future directions of this research will include examining the cellular mechanisms of the female‐specific enhancement of bone healing as well as assessment of factors that compensate for the loss of *Hdac3* in males. Likewise, use of class I Hdac inhibitors within tissue‐engineering strategies aimed at enhancing bone regeneration are also warranted.

## AUTHOR CONTRIBUTIONS

**Elizabeth K. Vu:** Data curation (equal); formal analysis (equal); writing – review and editing (equal). **Ismael Y. Karkache:** Data curation (equal); formal analysis (equal); writing – review and editing (equal). **Anthony Pham:** Data curation (supporting); formal analysis (supporting); writing – review and editing (supporting). **Elizabeth W. Bradley:** Conceptualization (lead); data curation (supporting); formal analysis (supporting); funding acquisition (lead); project administration (lead); writing – original draft (lead). **Jinsha Koroth:** Data curation (supporting); formal analysis (supporting); writing – review and editing (supporting).

## CONFLICT OF INTEREST STATEMENT

The authors declare no conflict of interest.

## Data Availability

The data that support the findings of this study are available from the corresponding author upon reasonable request.
